# Analysis of Accuracy in Pointing with Redundant Hand-held Tools: A Geometric Approach to the Uncontrolled Manifold Method

**DOI:** 10.1371/journal.pcbi.1002978

**Published:** 2013-04-04

**Authors:** Domenico Campolo, Ferdinan Widjaja, Hong Xu, Wei Tech Ang, Etienne Burdet

**Affiliations:** 1 School of Mechanical and Aerospace Engineering, Nanyang Technological University, Singapore; 2 Division of Psychology, Nanyang Technological University, Singapore; 3 Bioengineering Department, Imperial College of Science, Technology and Medicine, London, United Kingdom; University College London, United Kingdom

## Abstract

This work introduces a coordinate-independent method to analyse movement variability of tasks performed with hand-held tools, such as a pen or a surgical scalpel. We extend the classical uncontrolled manifold (UCM) approach by exploiting the geometry of rigid body motions, used to describe tool configurations. In particular, we analyse variability during a static pointing task with a hand-held tool, where subjects are asked to keep the tool tip in steady contact with another object. In this case the tool is redundant with respect to the task, as subjects control position/orientation of the tool, i.e. 6 degrees-of-freedom (dof), to maintain the tool tip position (3dof) steady. To test the new method, subjects performed a pointing task with and without arm support. The additional dof introduced in the unsupported condition, injecting more variability into the system, represented a resource to minimise variability in the task space via coordinated motion. The results show that all of the seven subjects channeled more variability along directions not directly affecting the task (UCM), consistent with previous literature but now shown in a coordinate-independent way. Variability in the unsupported condition was only slightly larger at the endpoint but much larger in the UCM.

## Author Summary

Daily motor tasks typically involve more degrees-of-freedom than strictly required. For instance, pressing a button in the elevator only requires positioning the fingertip at a three-dimensional location in space. However, to move the arm we need to control many more degrees-of-freedom (at least seven, only considering the shoulder, elbow and wrist) than required by the task, each with its own variability due to physiological factors such as tremor. Variability at proximal joints (e.g. shoulder or elbow) is expected to be amplified and projected at the distal end (fingertip). Remarkably, inter-joint coordination reduces the final variability at the fingertip position. Recent theories, such as the uncontrolled manifold (UCM), distinguished between inter-joint variability that would not affect the finger position and variability that would affect the final task. A major issue is that traditional UCM methods rely on the coordinate system chosen to analyse the arm motion. Therefore, we introduce a coordinate independent UCM method for tasks performed with handheld tools, e.g. surgery. This paper describes a new method and demonstrates that it enables an accurate analysis of static pointing. The results clearly show that the subjects can channel variability in dimensions that do not affect the task outcome.

## Introduction

Although highly stereotyped, human movements performed with the same intention are never exactly the same, displaying large variability in consecutive trials. Rather than just ‘biological noise’, many studies have pointed out how variability may in fact provide important clues on the underlying neural strategies. Analysis of structure in variability, and its changes, has therefore become an important tool for researchers in neuromotor control and learning, especially in presence of redundancy [1]. Here, we are interested in the analysis of accuracy of pointing tasks performed with hand-held tools, for applications such as surgery.

The problem of variability in redundant motor tasks was formulated by Bernstein [2], who studied the kinematics of skilled movements performed by professional blacksmiths while striking a chisel with a hammer. Bernstein observed how the variability of the trajectory of the hammer, at its tip, was in fact smaller than the variability of the trajectory of each of the joints of the arm holding the hammer. This suggests that the individual joints are not controlled independently and that the brain exploits kinematic redundancy to accurately control the endpoint.

Redundancy and motion variability are important not only for blacksmiths but characterize virtually every daily activity, from grasping a cup to signing off a letter, where we typically have many more degrees-of-freedom (dof) than necessary to fulfil the task. We are particularly interested in tasks involving hand-held tools such as microsurgery, where noise induced by tremor, amplified by the visual magnification provided by the optical microscope, is a critical factor of performance [3].

**Figure 1:**
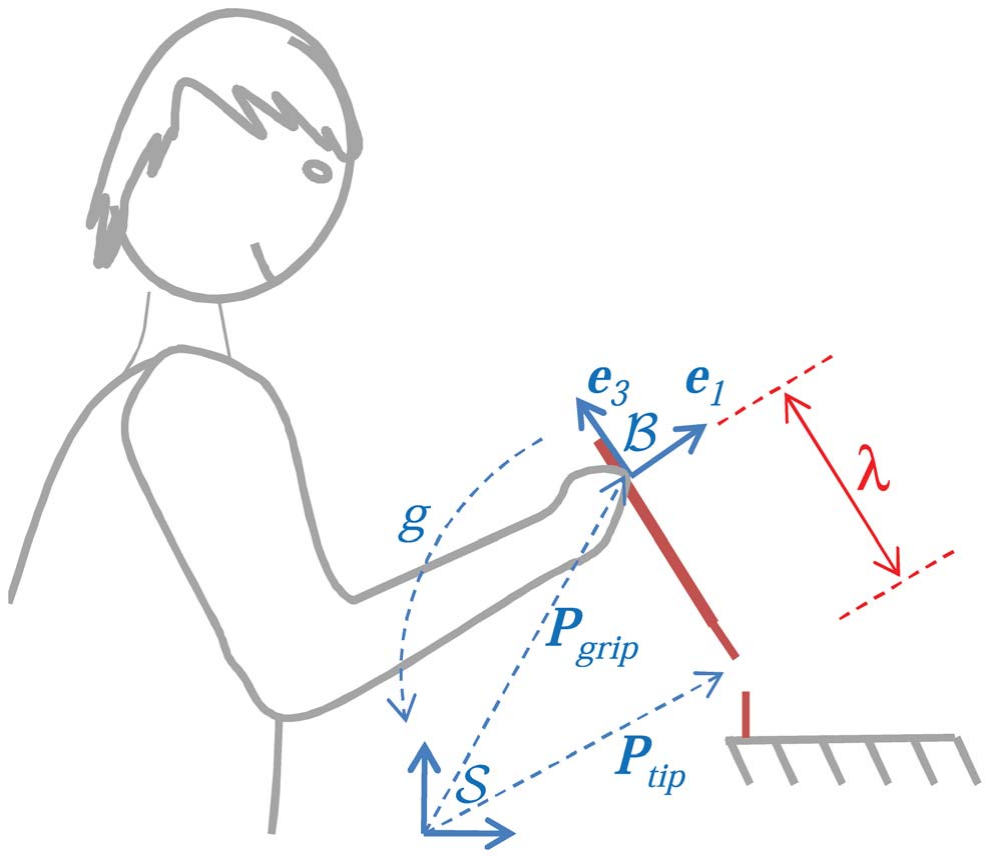
Static pointing task performed with a hand-held tool. The tool is grasped at a fixed position 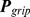, at distance 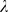 from the tool tip 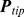. The pose 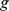 of the hand-held tool, with respect to a fixed frame 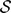, can be specified as the position 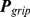 and orientation 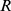 of a moving frame 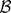 attached to the tool at the gripping point.

In this work, we consider static pointing tasks, such as the one in Fig. 1, where a subject is asked to keep the tip of a pen-like tool, e.g. a surgical scalpel, in steady contact with another object. In other words, the position of the tip, characterized by 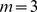 dof of mobility, is prescribed while the subject is free to choose among different postures, which include positioning of the torso, joint angles of the arm (i.e. shoulder, elbow and wrist) as well as grasping pattern of the hand. For postures away from biomechanical limits, there exists a *task-equivalent manifold* consisting of distinct postures which do not affect the task. A major challenge with real-world scenarios is that, due to the large number of degrees of freedom involved in the task, biomechanical analysis would be either intractable or oversimplified.

A key aspect of our study is that *the tool itself has redundant degrees of freedom with respect to the task*, i.e. subjects are asked to control position and orientation of the tool (6dof) while maintaining a steady tool tip position (3dof). On the one hand, this allows one to focus on the low-dimensional space of tool configurations, rather than the high-dimensional space of possible postures. On the other hand, by focusing on the tool, we can make use of the geometric properties of rigid bodies, as detailed in the following sections.

Scholz and Schoner [4] hypothesized that movement variance across task repetitions projects differently onto the task-equivalent manifold than it does onto the orthogonal complement directly affecting the task. A larger variance projected in the task-equivalent space (or nullspace) is indicative of neural control and the task-equivalent manifold (where larger variance is expected for skilled movements) was named uncontrolled manifold (UCM).

Despite its appeal, the computational procedures behind UCM (and principal component analysis in general) have been recently criticized for being coordinate-sensitive [5]. A fundamental issue with UCM is that the orthogonal space is typically defined via the standard Euclidean metric applied to the space of joint angles. Such an implicit choice is coordinate-dependent and the results of the UCM analysis would change if, for example, we decide to represent postures via joint angles instead of a normalized angle (e.g. the joint angle divided by the biomechanical range of motion).

More importantly, covariance-based analysis may reach different results if the coordinates are transformed. Even the linear transformation of joint angle coordinates from absolute to relative leads to different conclusions [5].

More than a century ago, Physics undertook a geometrization process of its main theories in the effort to achieve descriptions of phenomena in a coordinate-independent way, using differential geometry. Computational modelling in motor control is still at an early stage although some attempts have been made in this direction, e.g. [6], [7], [8], [9]. It is not clear which coordinate system might be adopted by the brain to represent postures, therefore any specific selection rests on an arbitrary choice of the researcher. In situations where the configuration space is the set postures determined by several joint angles, it is not clear how to introduce an appropriate metric structure which relates angles relative to different joints, with very different ranges of motion. For example, starting from a reference posture, can we say that two new postures achieved, respectively, via a 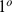 of ankle rotation and 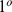 of knee rotation are equally ‘distant’ from the reference posture?

Although internal representations are largely unknown, it is clear that the brain does take into account the geometry and physics of the external world. Early studies on reaching tasks in the horizontal plane showed how we consistently move along straight lines in the extrinsic, end-point space [10]. In the last three decades, similar studies have been conducted under different conditions, including visual distortion [11] and force perturbations [12], producing evidence that the brain is able to learn and adapt in order to produce straight lines in the visually perceived space (so, sometimes slightly curved in the actual space). Computational studies showed how this large body of experimental observations is compatible with optimization of kinematic and/or dynamic costs which are related to the dynamics of the task, e.g. inertial and geometric properties of the human limbs [13], [14], [15]. These studies make use of methods traditionally applied in Mechanics and Robotics.

Furthermore, a very recent study by Danziger and Mussa-Ivaldi [16] supports the hypothesis that movement trajectories are based on the perceived geometrical properties of the object (such as the hand-held tool, in our paper) that is being controlled by the brain. The novelty of their approach is in the use of a virtual object controlled via the (hyper-redundant) posture of the fingers, an experimental paradigm designed to eliminate any bias due to limb dynamics and experience in reaching. The findings of Danziger and Mussa-Ivaldi [16], along with the work of Biess et al. [8], [9], suggest that *metric properties such as distance measures and geodesics (i.e. ‘straight lines’) of the operational space (be it virtual or real) play an important role in shaping our motor strategies*. In this sense, Riemmanian geometry represents the appropriate theoretical framework for analytical investigation in motor control.

In this paper we study accuracy during pointing via a coordinate-independent analysis of variance based on a choice of metric structure suggested by the specific application, in our case manipulation via hand-held tools. The paper outline is as follows. Next section will present all the steps involved in the classical UCM approach, based on vector calculus. Then an overview of the Riemannian geometric framework required to extend such vector calculus steps to more general settings is presented, along with the detailed formulation required to compute intrinsic variance based on metric properties of rigid bodies motions. This approach is then applied to the analysis of data of healthy subjects performing static pointing tasks. Results from the experiments are presented and discussed.

## Materials and Methods

### Classical UCM approach

The starting point for classical uncontrolled manifold (UCM) analysis is the definition of a forward kinematic model
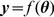
(1)relating 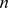-dimensional human joint variables 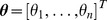 to a particular 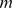-dimensional variable 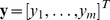 which is hypothesized to be directly controlled by the brain. For example, to investigate how the center of mass (COM) is controlled by children during quiet stance, Wu et al. [17] derived a forward kinematic model mapping seven joint angles (i.e. the angles formed between the foot, shank, thigh, trunk, head/neck, forearm, and upper arm segments with the horizontal) onto the anterior-posterior position of the center, a one-dimensional variable. The task is kinematically redundant as a 7D joint configuration space is mapped onto a 1D task space (anterior-posterior sway of the center of mass).

The UCM analysis is simplified by linearizing the nonlinear forward kinematics about the average posture, hereafter reference posture, computed across 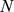 measurements:
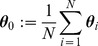
(2)Linearization around the reference posture via the 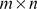 Jacobian 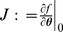, a matrix mapping joint-space velocities into velocities of the COM, is used to map ‘small variations’ in the joint space into ‘small variations’ in the controlled variable space:
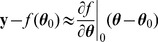
(3)As an approximation of the UCM, the nullspace (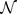) of the Jacobian is used, i.e. a linear subspace of the configuration space for which deviations from the reference posture produce no motion in the task space.
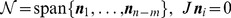
(4)At the same time a linear subspace ‘orthogonal’ to UCM (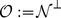) is also computed as the orthogonal complement to the nullspace
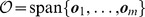
(5)Experimental deviations from the reference posture 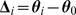 are projected onto the two orthogonal subspaces named, respectively, UCM (‘null’) and orthogonal (‘orth’) component.
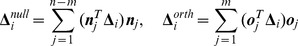
(6)Variance is computed for each component and normalized by the dimension of each subspace
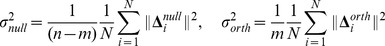
(7)where 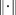 represents the Euclidean norm. The so called *UCM ratio* is then computed as the logarithm of the ratio of the variance of the UCM and orthogonal components.

### A Riemannian framework for intrinsic statistical analysis

Riemannian geometry allows generalizing to nonlinear spaces traditional concepts and tools from vector calculus, e.g all the steps behind the classical UCM approach. In this section, we shall try to build some intuition to help relating these new geometric tools with the classical ones. For a more comprehensive and detailed description, the reader is referred to [8], [9] and references therein. To motivate this need of generalization, one can think of cartography: our rigid rulers and goniometers work well on flat sheets of papers but not on a globe. Therefore cartographers draw charts by mapping points on the globe onto a sheet of paper. Clearly, patching the globe requires multiple charts, each with its own coordinate system (‘squared’ paper), and some rule to reconcile overlapping charts.

**Figure 2:**
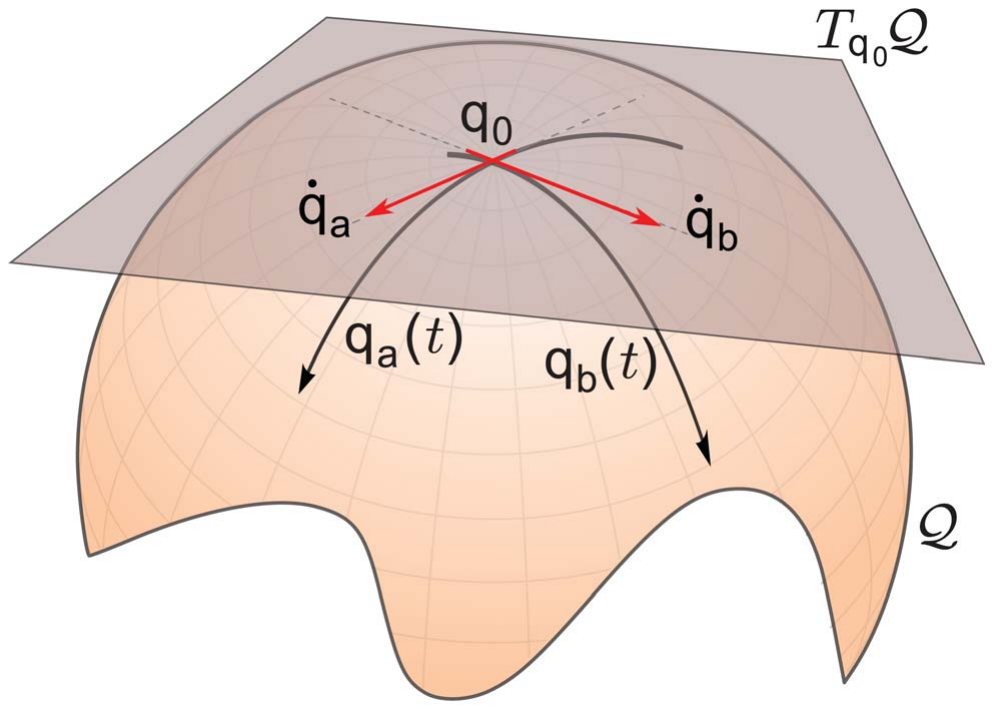
Example of configuration manifold.

With reference to Fig. 2, the starting point is to define a 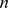-dimensional *configuration manifold* (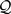) and to patch it with coordinate charts. Any chart of coordinates 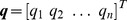 is sufficient to describe the behavior of the system *locally*, i.e. around a reference point (or posture) 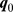 in the configuration manifold. Around such a reference point, we can approximate the manifold with its *tangent space* (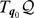), an 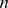-dimensional linear (vector) space, tangent to the manifold at the reference position. The tangent space will be also tangent, at 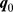, to any trajectory 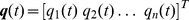 on the manifold passing through 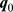 itself. For this, elements of the tangent space are naturally identified with velocity vectors 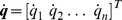, at 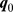.

The central element in Riemannian geometry is the introduction of a *metric*, i.e. a symmetric, positive definite bilinear function (or quadratic form) 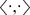. As a generalization of the inner product of Euclidean spaces, a Riemannian metric acts on pairs of vectors (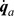, 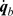) tangent to the manifold at a given point to determine, for example, the length of a vector (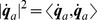) or the angle 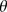 between them (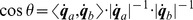).

#### Kinetic energy metric

A metric does not come with the manifold, it is extra structure that should be defined based on the application. In this lies the connection to Mechanics: *the kinetic energy of a system naturally defines a metric*. For a very general class of mechanical systems, including the ones of interest in Robotics, the kinetic energy (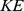) can always be expressed as
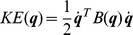
where the configuration-dependent matrix 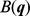 is defined based on the inertial and geometric properties of the system and can be shown to be always symmetric and positive definite and can be used to define a bilinear metric form as
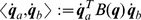
Once a metric structure is in place, vector calculus methods can be generalized for use on general manifolds *independently of the choice of coordinates used for the analysis*. In the following, we should examine only those which will be used in this paper.

#### Length of trajectories

if our manipulator moves from an initial posture 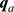 to a final posture 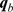 via a trajectory 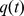, the length of the curve connecting the initial and final postures is
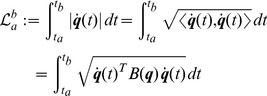
(8)


#### Geodesics

among all the possible smooth trajectories between two points, the geodesics are the ones of minimum length in the sense of eq.(8). Geodesics are found by solving a second order differential equation which involves first derivatives of the metric coefficients (corresponding to the Coriolis' and centrifugal terms in the standard equations of manipulators). We are not solving it explicitly as we will use results from other works, so we refer the reader to Biess et al. [9] for further details. What really matters here is that, from a given point 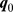 only one geodesic exists which passes through it with a prescribed velocity 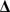, at 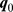.

There is an important map from the tangent space to the manifold itself which is known as *exponential map*:
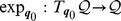
A manipulator with an initial configuration 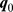 and initial velocity 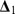, if unperturbed by external forces, after a unit time would evolve into a new posture 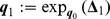.

There is also its inverse map, known as *logarithmic map*

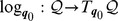
A manipulator with an initial posture 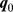 will evolve, in a unit time and without external perturbations, into a final configuration 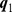 only if the initial velocity is 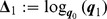. The logarithmic map extends to Riemannian manifolds the concept of *difference* between two vectors, which only makes sense on linear spaces.

#### Distance between two postures

if reaching a new position for a starting point in a unit time requires a large initial velocity, despite the choice of the shortest path, then the two points are probably far apart. The initial velocity, for a geodesic, is therefore a good measure of *distance*. The usefulness of the logarithm (at least locally) is in the ease of computing the distance between two points
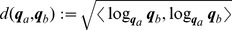



#### Intrinsic mean [18]

Given a set of 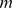 postures 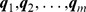, how can we define a *mean posture*? This is one very clear example where using vector calculus leads to coordinate-dependent results. Once a Riemannian framework is in place, one can define an intrinsic, i.e. coordinate-independent, mean posture as
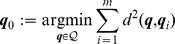



#### Intrinsic variance [18]

Once we have a mean value 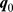, we can consider the tangent space 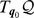 and project onto it all the samples 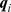 via the logarithmic map:
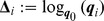
As previously mentioned, the logarithm 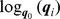 can be thought of as *difference* between 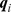 and 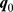. It should also be noted that, by definition of the logarithm, 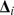 is an element of the tangent space, i.e. a vector, and as such we can easily extend classical formulations for an intrinsic definition of variance
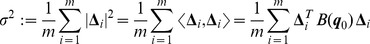



### Rigid body motions: a metric for hand-held tools

As mentioned previously, a metric does not come with a configuration manifold, it is extra structure which is typically defined by the application. Therefore *the investigator always has to make a choice*, especially when defining experimental conditions. Once the choice is made, the Riemannian geometric framework ensures that the results of the analysis will not depend on the choice of coordinates.

The general Riemannian geometric approach starts from the definition of the configuration manifold. When it comes to rigid body motions, the configuration manifold is more structured than the general case. The space of rigid body configurations is in fact a *Lie group*, a manifold with additional algebraic structures as we shall see next.

With reference to Fig. 1, consider a space-fixed frame 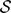 and a moving frame 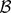 attached to the hand-held tool at the gripping point. Let 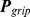 represent the 3D coordinates of the gripping point in space coordinates. The orientation of 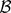 with respect to 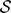 is determined by the coordinate axes 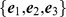, the latter being aligned with the tool major axis, pointing away from the tip. At all times, the orientation of 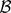 relative to 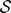 can be represented via a 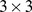 rotation matrix 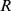 whose first, second and third columns represent, respectively, the space-fixed coordinates of the axes 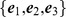.

#### Rigid body rotations

The 3D orientation of a rigid body can be described by means of a 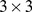 rotation matrix 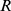 (satisfying ortho-normality 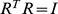 and right-handedness 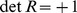). A rotation is physically determined once the rotation axis 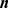 (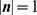) and the rotation angle 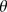 are known, thus can be described by a rotation vector 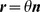.

The rotation matrix 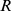 corresponding to a rotation vector 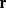 can be computed via the *exponential map* [19]:
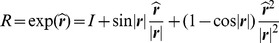
(9)where the skew-symmetric matrix 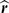 is defined via the ‘hat’ operator:
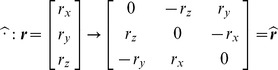
(10)Conversely, for a given rotation matrix 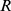, the corresponding rotation vector can be computed via the *logarithmic map* [19]:
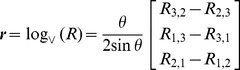
(11)where 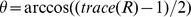, valid for 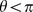. As a note, the classical definition of logarithm (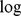) of a rotation matrix returns a skew-symmetric matrix, which is associated with a unique 3D vector via the natural isomorphism eq. (10). The map 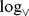 simply combines the logarithm and the isomorphism, returning directly a rotation vector.

#### Body angular velocities

For a rigid body pivoting about a hinge, 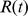 represents the trajectory in the configuration space and 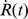 is the instantaneous velocity, as seen previously for general manifolds. However, from Physics, we expect a three-dimensional body velocity while 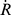 requires nine coefficients (obviously correlated). The reason is that 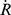 maps the body positional coordinates into the spatial velocity coordinates. It can be shown [19] that expressing the same velocity in body coordinates leads to skew-symmetric matrix, corresponding to the usual definition of *body angular velocity*

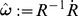



#### Rigid body motions

The *pose* of a rigid body, e.g. the hand-held tool in Fig. 1, is fully specified by the relative position 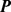 and orientation 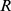 of the moving frame 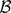 with respect to the space-fixed frame 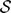. The pose 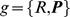 can be conveniently represented as a 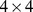 homogenous matrix
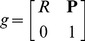
(12)The velocity 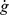 of the rigid body from the perspective of the moving frame (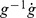) can be concisely written as a 6D *generalized body velocity* vector
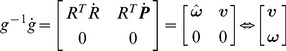
(13)which contains the body linear velocity 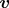 and body angular velocity 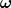. Dually, one could define generalized space velocities via 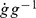.

#### Changing frames

If we were to describe a physical motion 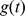 with respect to a new spatial frame, displaced from the original one by a constant matrix 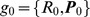, we would obtain a new description 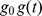 for the *same* physical motion. In general, this would lead to a new velocity reading 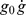 but it would *not affect body velocities* since
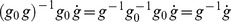
(14)As a change of spatial frame corresponds to *left*-multiplying by a constant matrix 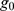, invariance to this transformation is called *left-invariance*. Dually, a change of moving frame corresponds to a right-multiplication (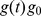) and invariance to this transformation is called *right-invariance*.

#### Left-invariant kinetic energy metric

The kinetic energy (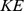) of a rigid body is a scalar quantity which clearly cannot depend on the frames (moving or spatial) used to describe the kinematics of the object. As known from basic Mechanics, when the moving frame is located at the center of mass of the rigid body, the kinetic energy can be conveniently written in body coordinates as
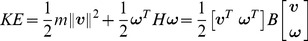
(15)with the generalized inertia matrix (relative to the center of mass) being:
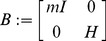
(16)where 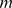 is the mass of the rigid object, 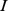 is the 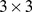 identity matrix and 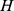 is the moment of inertia. Since body velocities are by definition independent of the spatial frame, as shown in (14), general invariance of the kinetic energy implies that also the generalized inertia matrix (16) must be invariant to a change of spatial frame, i.e. left-invariant. A *left-invariant Riemannian metric* can then be defined from (15) as
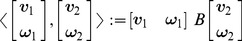
(17)It is worth noting that the metric (17) is not invariant to changes of moving frame, i.e. *not right-invariant*, as the generalized inertia matrix 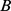 strongly depends on the moving frame. The formulation in (15) is in fact only valid when the moving frame is located at the center of mass. This reflects an important property of rigid bodies: the *lack of a bi-invariant metric*, i.e. a metric which is independent of both the spatial and the moving frame [20].

In dynamics, the inertia matrix can be determined once the shape and the material properties such as density of an object are known. Clearly, when kicking an object, it makes a lot difference for the subsequent motion whether it is a round soccer ball or an elliptical rugby ball.

However, for kinematic purposes, inertial properties may not be of interest. For example, in our application, if the hand-held tool is very lightweight and our motion is relatively slow, its shape and material properties might be neglected at a first level of analysis. In such cases, we can abstract the inertial properties and consider an isotropic inertia, rather than a general ellipsoid, by setting 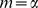 and 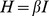, where 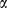 and 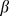 are scalars
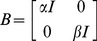
(18)This metric was introduced by Park and Brockett [20] as the *scale-dependent left-invariant metric* for rigid body motions. The two parameters 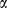 and 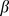 weigh the relative importance of rotations versus translations. As highlighted by Park [21] there is no natural choice for these scalar coefficients and a selection will depend on the application. In our case, any scaling factor common to both 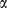 and 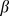 would not change the orthogonality conditions nor the ratio of variances, so we can fix 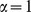 and define a value for 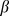. As evident from Fig. 1 and detailed in next section, an infinitesimal rotation 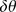 at the gripping point about 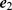 would cause a tip displacement 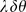 along 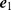. This suggests setting 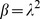, i.e.
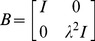
(19)and the inner product reduces to
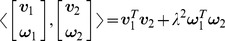
(20)As shown by Park [21], [22], based on the metric defined by (19), the geodesic curve 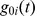 connecting, in a time unit, an initial pose 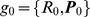 to a final pose 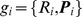 is
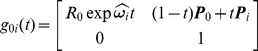
(21)where 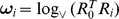. The ‘difference’, in a Riemmanian sense, between the two poses can be identified with the body velocity of the geodesic motion
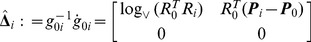
(22)It is worth noting that, for the metric (18), geodesics evolve at constant body velocity 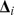 [23]. The distance between the two poses is therefore
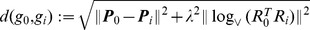
(23)Once a metric is in place, one is actually able to relate different tangent spaces via the so called ‘metric compatible connection’ and perform, for example, proper differentiation of velocity vector fields which leads to a correct definition of acceleration [23].

### Left-invariant kinematics for the pointing task

The focus of our analysis is on pointing tasks performed via hand-held tools. The key aspect is that *the tool itself has redundant degrees of freedom with respect to the task*. This allows disregarding the complexity of the body postures (which especially for modeling the grasping finger pattern would either lead to oversimplified models or intractable ones) and focusing on the intermediate space of tool configurations. In fact, we can think of the forward kinematics as a concatenation of two maps: the first transforming bodily postures to tool poses, the second transforming tool poses to controlled variables, i.e. the tool tip. To determine the latter map, we should consider that the gripping point of the hand tool is at a constant distance 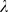 from the tip, therefore
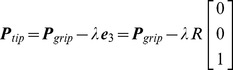
(24)Differentiating with respect to time leads to
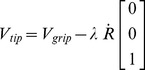
(25)where 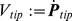 and 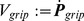 are, respectively, the velocities at the tip and at the gripping point, in space coordinates.

As we seek a formulation which is spatial frame invariant, we will try to express everything in body coordinates. Body velocities 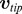 and 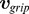 are related to space velocities via the transformations 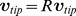 and 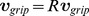, which leads to
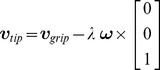
(26)where 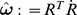 is the skew-symmetric matrix for the body angular velocity. Equation (26) is clearly invariant to changes of space frame as it only comprises constants and body velocities which are left-invariant, as shown in (14). A more compact formulation in terms of generalized velocity leads to
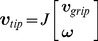
(27)where 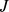 is the Jacobian matrix
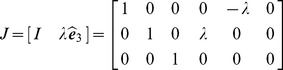
(28)In the kinematic relation (27), the Jacobian projects the 6 dof generalized velocity of the hand-held tool into the 3 dof velocity of the task. This captures the redundancy of the tool with respect to the prescribed task.

### Null and orthogonal spaces

The nullspace 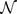 is the sub-space of generalized velocities 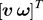 of the tool which produce no motion at the end-tip, i.e. 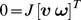. It is straightforward verifying that the following generalized velocities
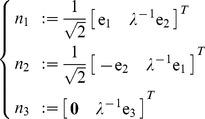
(29)are an orthonormal basis of the nullspace of 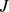, i.e. produce zero end-tip velocity 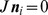 for 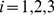; are mutually orthogonal 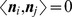 whenever 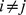; and have unitary length 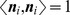 for 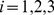.

Similarly, it can be verified that
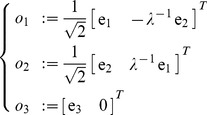
(30)is an orthonormal basis of the orthogonal complement of 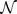


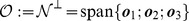
(31)


### A reference position for hand-held tools

Given a set of 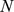 rigid body poses 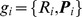, where 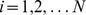, an *intrinsic* definition of mean pose, see [24], [22] and reference therein, is
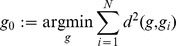
where 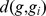 is the distance between the poses 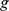 and 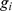 as in (23). It can be shown that the intrinsic mean pose can be expressed as 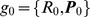, where
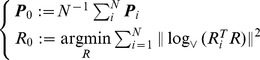
(32)For further details, the reader is referred to [24], [22].

### Analysis of variance via principal geodesic analysis

In the classical UCM approach [4] [17], where postures are typically parameterized via a vector of 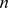 joint angles 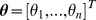, deviations from the reference position 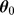 are directly computed as differences between vectors 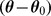 and projected onto the nullspace of the Jacobian and onto its orthogonal complement.

For nonlinear spaces as for rigid body motions, this is not possible and we will extend the classical UCM approach with the concept of geodesics as proposed by Fletcher et al. [24]. Starting from a given point (e.g. a reference position), geodesic curves are completely specified once the initial velocity is given and allow connecting sufficiently close points via minimal paths. Therefore, geodesics are a natural way to define the deviation of a point B from a point A as the initial velocity for a geodesic curve to start in A and reach B in a unit time.

Recalling (22), the ‘difference’ between a pose 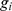 and the reference pose 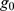 can be determined as the (constant) body velocity required to join the two poses via a geodesic in unit time:
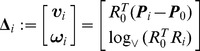
(33)By definition, the body velocity is a vector of the tangent space and can be projected onto the UCM and its orthogonal complement via the available inner product (20):
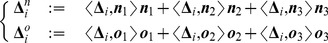
(34)As in the classical UCM approach, variance-per-dof can be computed as
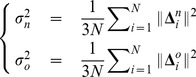
(35)where 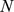 is the number of measurements and 3 is the dimension for both the nullspace and its orthogonal complement. The definition in eq. (35) corresponds to the geometric framework proposed by Fletcher et al. [24] where (based on the early work of Frechet [25]) the variance of a random variable in a metric space is defined as the expected value of the squared distance from the mean.

This section first introduced the general steps involved in the classical UCM approach and then derived an intrinsic definition of each of these steps for the case of static pointing with hand-held tools. In particular, an intrinsic definition of deviations eq. (33) from an average pose eq. (32) was constructed by means of geodesics. In this way, the variance eq. (35) of these deviations on nullspace and on its orthogonal complement can be carried out independently of the choice of coordinates.

### Experimental setup

To analyze variability during static pointing tasks with hand-held tools, experiments were conducted with 7 healthy subjects without any known history of neuromuscular impairment. All of them declared to be right-handed and gave their informed consent prior to the experiment. The study was approved by the institutional review board of Nanyang Technological University and was conducted according to the principles expressed in the Declaration of Helsinki. Each subject was asked to hold a sensorized stylus of a Polhemus Liberty system (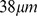 and 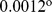 resolution within 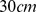 range) at a specific gripping point 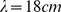 from the tip, onto which a hypodermic needle with luer connector (Terumo 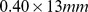) was attached. The subject was then asked to touch the tip of a similar needle, firmly attached to a wooden table in a vertical position, with the tip of the stylus, as shown in Fig. 1. The position of the tip 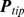 and the orientation of the stylus 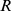, which are related to the position of the gripping point 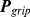 via eq. (24), were acquired at 240 Hz via the Polhemus Liberty system and recorded onto a local PC for off-line data analysis. For both needles, only 1 mm of the tip is exposed while the remaining part is isolated with tapes. The setup is such that a beeping sound is produced when electrical contact between the exposed tips of the two needles occurs.

### Experimental protocol

**Figure 3:**
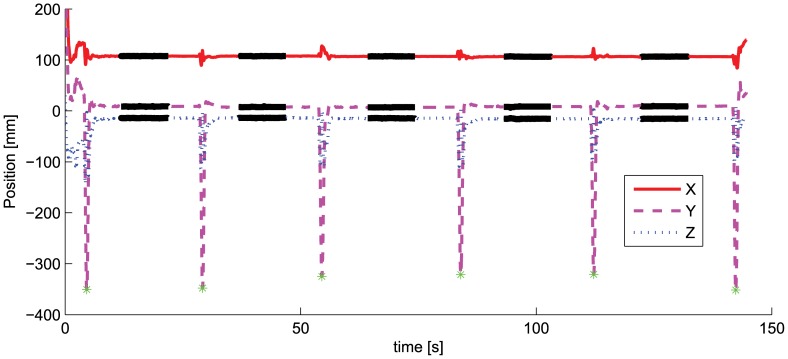
Components of the 3D position, in spatial frame coordinates, of the stylus tip (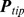) for one representative trial. The ‘ * ’ marking the minima correspond to the positions furthest away from the target. The thick solid lines are the data of interest, within 5 seconds before and after the midpoint between two minima.

The experimental protocol consisted of 20 consecutive trials. In each trial, the subject was asked to make a 15 seconds, steady contact between stylus and target tips separated each time by a large movement of the stylus approximately 20 cm away from the body. Only the inner most 10 seconds between two large movements were analyzed (thick solid lines in Fig. 3). Firstly, the furthest positions away from the target were detected (corresponding to minima in Fig. 3) then a midpoint was calculated. For data analysis we considered only the data points within 5 seconds before or after the midpoint.

One minute rest was given every 5 trials. No visual magnification was provided to the subject. The protocol was performed in two different experimental conditions: in Exp I the elbow of the right arm was supported on the table, while in Exp II the arm was unsupported, resulting in different noise conditions [26]. For every subject, both types of experiments were carried out in the same day, with Exp I preceding Exp II and one hour rest in between. The data relative to the three components of the tip position (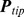) during one representative trial are shown in Fig. 3.

### Data reduction

For each trial, only the inner most 10 seconds (2400 samples) of steady contact were analyzed.

To detect physiological tremor, the power spectral density (PSD) was estimated. To this end, the velocity components along each axis were estimated by numerically differentiating the tip position, component-wise. Then, for each (10 seconds) trial, the pwelch() function in the MATLAB environment was used to estimate the average PSD over ten non-overlapping time windows (1 second each). Finally, for each subject, the PSD estimates obtained for each trial were averaged.

**Figure 4:**
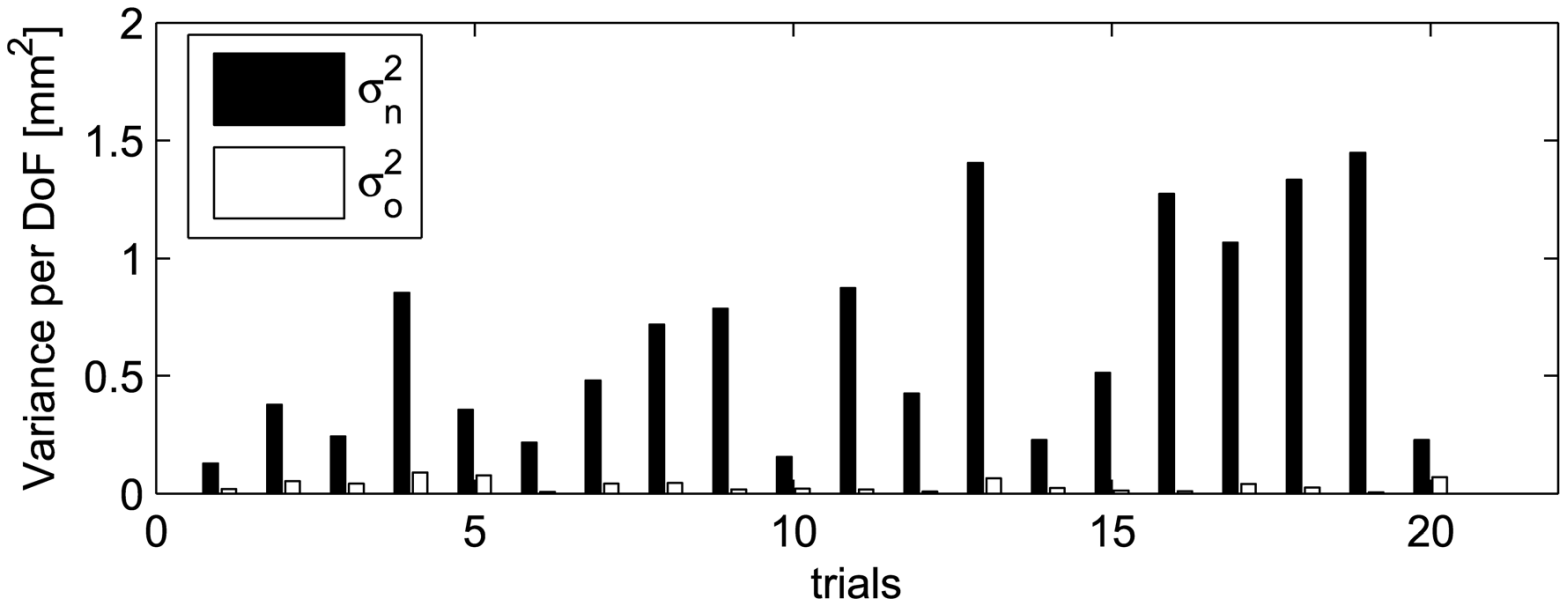
Variance per dof in the nullspace (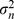) and its orthogonal complement (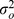) for all 20 trials of a representative subject.

For each trial, the relative UCM components 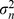 and 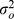 were computed as in eq. (35). The logarithm of their ratio, referred to as *UCM ratio*, was computed
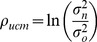
(36)The logarithm, instead of the ratio, was used in order to correct for non-normal distribution [27], [28]. The UCM components for all trials of a representative subject are shown in Fig. 4.

Similarly to the UCM components and their ratio, also the variances of 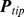 and 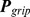 and their ratio
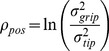
(37)were computed for each trial.

### Statistical analysis

According to the UCM theory [4], a larger variance in the null space (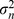) than in its orthogonal complement (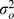) indicates that the position of the stylus tip is a variable directly under neural control. Therefore, we hypothesized that the UCM ratio (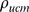) will be significantly greater than zero. We also tested the influence of the experimental conditions (Exp I and Exp II) on the UCM ratio. Similar analysis was conducted for the variances of 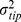, 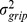 and their ratio eq. (37).

To test whether the average UCM ratio eq. (36) is significantly different from zero, a Wilcoxon signed rank was run for the each subject on the 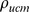 values derived from every trial, separately for the two experimental conditions Exp I and Exp II. Similar tests were conducted for 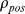.

A series of analysis of variance (ANOVA) tests with repeated measures was conducted to test the effect of experimental conditions and of UCM component on the variance-per-dof. The dependent variables are variance-per-dof, 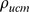 and 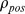. A three-way repeated measures ANOVA–2 (experimental condition) 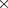 2 (UCM component) 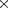 20 (Trial) was conducted on variance-per-dof. Two 2 (Experimental condition) 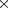 20 (Trial) repeated measure ANOVAs were conducted on 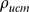 and 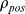. All the ANOVAs used the MATLAB implementation RMAOV33 [29] and RMAOV2 [30]. A series of Kruskal-Wallis test were conducted to test the effect of experimental conditions on 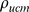 and 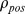 for every individual subject.

## Results

**Figure 5:**
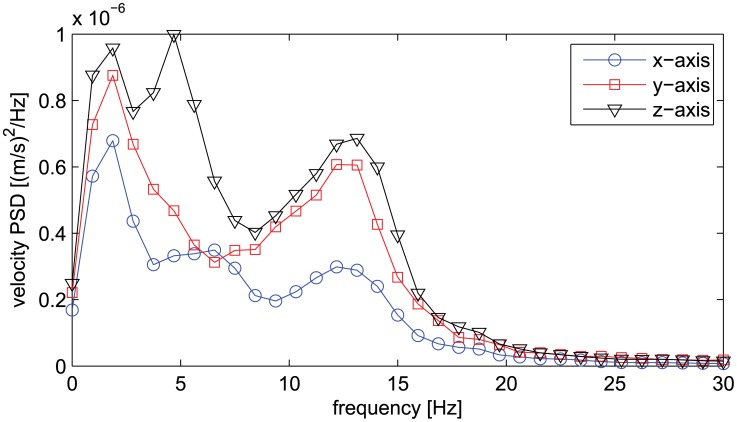
Power spectral density for each velocity component of a representative subject.

Analysis of the power spectral density of the variability of motions, revealed a frequency profile containing at least two major peaks in the frequency ranges 0–7 Hz and 7–15 Hz, consistent with the spectral density of physiological tremor [31]. Fig. 5 shows the averaged PSD for a representative subject.

### UCM components and ratio

**Figure 6:**
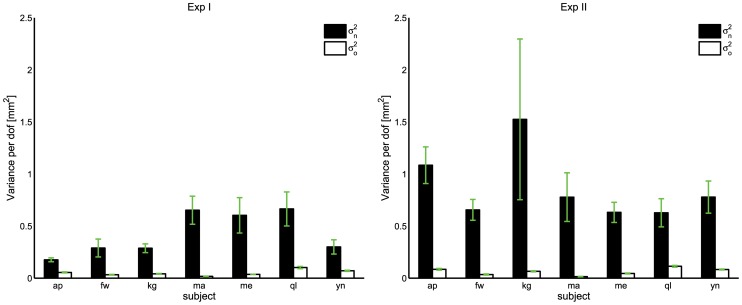
Average UCM components for each subject in Exp I (left, supported arm) and Exp II (right, unsupported arm). For both experimental conditions, each subject shows a statistically significant difference (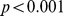) between the UCM components. Vertical lines represent the standard errors.

For both experimental conditions, there was much more variability in the UCM subspace than in the orthogonal subspace (i.e. 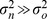, Fig. 6) as a Wilcoxon signed-ranks test showed a UCM ratio significantly different from zero (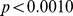).

A three-way repeated measures ANOVA (experimental condition 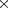 UCM component 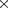 trials) conducted on the variance-per-dof indicated that all the main effects are significant: 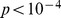 for the UCM component effect, 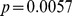 for the trial effect) and 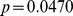 for the experimental condition effect. In addition, significant interactions were found between trial numbers and UCM component (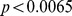 for all possible interactions).

**Figure 7:**
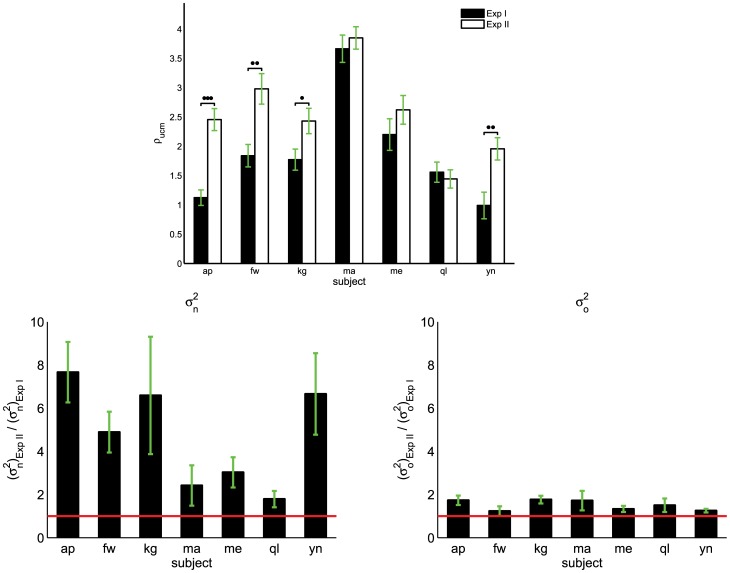
UCM ratio for seven subjects for the two experimental conditions, Exp I and Exp II (top). Bottom figures show the 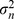 ratio between Exp II and Exp I (left) and similarly for 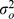 (right). The horizontal line marks the unitary ratio (i.e. no difference in the variance in position between Exp II and Exp I). Vertical lines represent the standard errors. Statistically significant differences are highlighted for 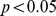 (*), for 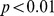 (**), and for 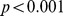 (***).

Effects due to experimental condition (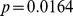) and trials (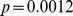) were also found in a two-way repeated measures ANOVA on 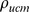 (Experimental condition 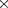 trial). No interaction effect was found between experimental condition and trial (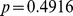). As shown in Fig. 7, there is a significant difference for the UCM ratio evaluated for the two experimental conditions in four subjects (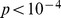, 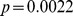, 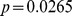, and 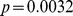, Kruskal-Wallis test on subject 1, 2, 3, and 7).

### Variance of position

**Figure 8:**
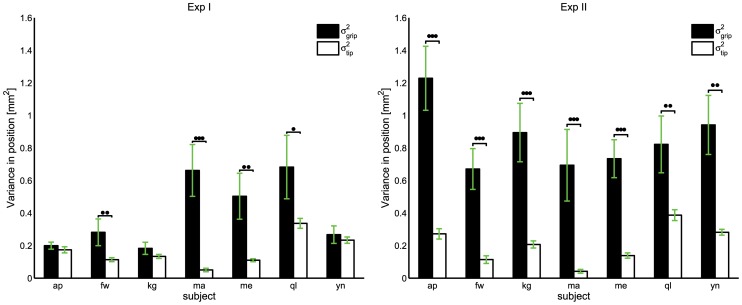
Variance at 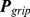 and 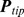 in Exp I (left) and Exp II (right). Vertical lines represent the standard errors. Statistically significant differences are highlighted for 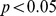 (*), for 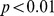 (**), and for 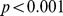 (***).

Similar analysis as for the UCM components and ratio, revealed, in general, more variability in 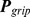 than in 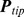, although this is not the case for all subjects (Fig. 8). Wilcoxon signed-rank tests on the 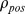 showed that all the 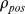 are significantly different from zero (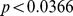) for all subjects and two experimental conditions, except subjects 1, 3, and 7 in Exp I (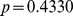, 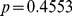, and 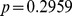 respectively). This indicates that for all subjects, the variance is larger at the gripping point of the hand tool than at the tip of the pen when the arm can move freely without support; while half of the subjects have similar variance at the gripping point and the tip when the arm is supported.

**Figure 9:**
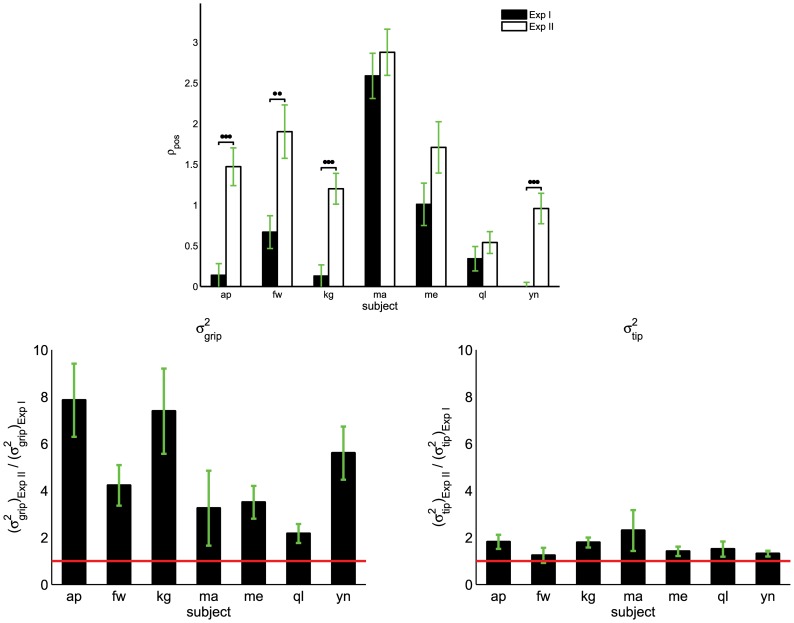
Ratio of position variance ratio (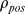) for each subject in both experimental conditions (Exp I and Exp II) (top). Bottom figures show the 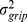 ratio between Exp II and Exp I (left) and similarly for 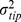 (right). The horizontal line marks the unitary ratio (i.e. no difference in the variance in position between Exp II and Exp I). Vertical lines represent the standard errors. Statistically significant differences are highighted for 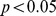 (*), for 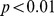 (**), and for 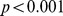 (***).

As shown in Fig. 9, a series of Kruskal-Wallis tests were applied to the 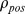 between two experimental condition and for subjects 1, 2, 3, and 7 there was a significant difference due to the experimental condition (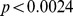), for the remaining subjects 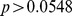. A two-way repeated measures ANOVA conducted on 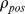 indicated that there were experimental condition effect (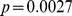) and trial effect (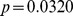) but no significant interaction (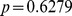).

## Discussion

### Motor redundancy: a blessing or a curse?

Complexity of the human body typically leads to an excess of degrees of freedom for virtually every motor task we are routinely involved with. Redundancy is also adopted in the design of artificial systems, e.g. articulated robots, as extra dof can increase dexterity and robustness. However, redundancy also requires sophisticated control strategies, for example in devising control laws which guarantee repeatable postures [32]. In this lies one of most fascinating aspects of human motion: the apparent conflict between repeatability and variability of the movement itself.

From an analytical perspective, repeatability and variability of movement have traditionally been distilled from experimental data via statistical approaches, by computing average and standard deviation estimates of movement properties derived from repeated trials. As pointed out by Newell and Slifkin [33], the vast majority of motor control literature on normal human subjects has neglected movement variability, considering it as a reflection of ‘biological noise’, while literature on motor disorders would interpret ‘low’ variability as a deficit, e.g. as in the case of stereotypies. Thresholds for standard deviations according to which the amount of variability should be considered large or small are often unreported, reflecting a bias relative to the theoretical views of the investigator. Furthermore, there is clearly more to movement variability than just standard deviation.

In the last decade, various researchers have started exploring the structure of variability rather than just its amount. Structure in variability has been so far explored along two major avenues: its temporal or its geometric features. These two aspects are by no means exclusive and, in general, a combined temporal and geometric analysis is likely to provide more insight into human motor control.

In this paper, we considered a pointing task and we focused on the geometric structure of variability, and also estimated the power spectral density to verify the frequency signature of physiological tremor. Consistent with previous literature on physiological tremor [31], our spectral analysis confirmed, for all subjects, the presence of at least two peaks in the frequency ranges 0–7 Hz and 7–15 Hz. However, variability is not just tremor, in particular in goal directed tasks where voluntary control actions are expected to take place. Furthermore, despite being a static pointing task, temporal aspects are still present due to, for example, fatigue and learning effects. In our analysis of variance, a main effect of trials was always present. Given the simplicity of the task, we believe that fatigue rather than learning might have contributed to the trial effect.

The geometric structure of variability was underlined by the pioneering work of Scholz and Schoner [4] who hypothesized that variability in redundant tasks is largely restricted to a subspace (UnControlled Manifold, UCM) of the configuration space which does not affect the task. Along this line, other related approaches have been proposed such as the Goal-Equivalent Manifold (GEM) [34] and Tolerance, Noise and Covariation (TNC) method [1]. While it is expected that the variability of the distal segments increases with the degrees of freedom, Morrison and Newell [31] showed that the contribution of more proximal upper limb segments to distal (finger) tremor is not simply additive, which is compatible with the hypothesis of neurally-driven compensatory strategies. In their study, Morrison and Newell [31] asked healthy subjects to minimize motion at the tip of the index fingers while standing with the arms parallel to the ground, with both index fingers fully extended and the remaining fingers fully flexed. The contribution of different joints to the distal tremor was analyzed by successively increasing the support of upper-limb segments from proximal to distal. Among other things, their study highlighted how the synergistic action of the wrist joint resulted in significantly smaller tremor at the index finger during a postural task.

Similarly to Morrison and Newell [31], we evaluated variability at a distal endpoint in two different conditions: supported (Exp I) and unsupported arm (Exp II). In addition, our task was designed to be kinematically redundant as we wished to analyze changes in terms of ‘good’ and ‘bad’ variability, where ‘good’ refers to the variability which does not affect the task (i.e. in the null space) and ‘bad’ denotes the variability directly reflected in the task space (i.e. in the orthogonal complement to the null space). As expected, in both experimental conditions, our results show that each subject shows a statistically significant difference (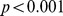) between the UCM components, projecting more tremor along directions which do not affect the task. Furthermore, in the unsupported-arm condition (Exp II), indeed more variability is introduced at the distal endpoint but that it is mostly channeled into ‘good’ variability (Fig. 6). This is also clear from Fig. 7 where the increase in both ‘good’ and ‘bad’ variability is analyzed when the experimental condition is changed from supported-arm (Exp I) to unsupported-arm (Exp II). While, in terms of mean values, ‘bad’ variability increases but does not double for all subjects, ‘good’ variability increases significantly for most of the subjects (for half of them, there is a five-fold increase).

We performed a similar analysis comparing variability at the gripping point and at the tool-tip. Although leading to qualitatively similar results, UCM analysis leads to ‘crisper’ results in terms of statistical significance. This was expected since the goal of the task, clearly defined in pointing tasks, is fully captured by the UCM analysis.

### A new tool to analyze skill

One of the most appealing aspects of the UCM method is the possibility to distinguish ‘good’ variance (not affecting the task success) from ‘bad’ variance (affecting task performance) and, therefore, to identify skillful performance. Subjects who are able to channel physiological tremor into movements which do not affect the task, might be deemed more skillful. Therefore one might be tempted to relate skills to the UCM ratio. However, our results suggest another possible explanation.

From our experiments, there appear to be two groups of subjects: those who show a statistically significant improvement in terms of UCM ratio (Fig. 7-top) in relation to a change of experimental condition and those who do not. A possible interpretation is that, while all subjects perform well in the unsupported-arm condition, the former group (formed by subjects ‘ma’, ‘me’ and ‘ql’) performs equally well also in the supported-arm condition. The latter condition is characterized by a reduced number of redundant degrees of freedom. In this sense, when more dof are available, it might be easier to channel variability into motions which do not affect the task (‘good’ variability) and, thus, skill might be related to the ability of performing equally well with a reduced number of dof. However, the mechanism behind this group difference is unknown and this requires further investigation.

### Coordinate independence and objectivity

Despite its appeal, a weakness in the UCM analysis has been recently pointed out in relation to its coordinate dependence [5]. An issue with UCM is that the orthogonal space is usually defined via the standard Euclidean metric applied to the space of joint angles. This choice is coordinate-dependent, thus the results of the UCM analysis would change if, for example, we decide to represent postures via joint angles instead of a normalized angle (e.g. the joint angle divided by the biomechanical range of motion).

As also mentioned by Sternad et al. [5], in computational motor control a distinction should be made between *internal* coordinates, which are assumed to be used by the brain to process information as well as plan and execute actions, and *external* coordinates used to describe and analyze behavior by the investigator. In the latter case, care must be exercised to ensure that the results are independent of the researcher's choice of coordinates.

Differently from the UCM approach, Sternad et al. [5] proposed a method where variability is evaluated in the “space of the result”, a task-related space, making it less sensitive to coordinates in the configuration (or execution) space. This is done in recognition of the fact that for unambiguously defined tasks there should be a natural way to evaluate performance, possibly leading to a well-defined metric in the task-space.

From this perspective, a main contribution of this paper is the use of task-specific features to construct an appropriate metric, which leads to a frame-invariant and objective analysis in the sense of [35].

In particular, manipulation via hand-held tools suggests the use of the scale-dependent left-invariant metric (18), a particular type of kinetic energy metric especially suitable for kinematic rather than dynamic analysis, initially proposed by Park and Brockett [20], [21]. Left-invariance guarantees independence of the inertial frame but the lack of a bi-invariant metric [21] implies dependence on the choice of body-fixed frame. Nevertheless, in the case of kinetic energy metrics for rigid body motions, left-invariance is in fact *sufficient to guarantee the principle of objectivity* [35].

From a mathematical perspective, it should be noted that one could have chosen a right-invariant metric to guarantee body-fixed frame indifference and forgo the left-invariance, i.e. bearing a dependence on the spatial frame. This would not be acceptable in our case, as the results would be dependent on the choice, for example, of the measuring system.

Previous mathematical arguments are very general and do not take into account the specific requirements of the task. To describe the pose of a tool, the experimentalist needs to choose two coordinate frames: a fixed frame 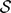 and a moving frame 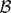. Our left-invariant description does not depend on the fixed frame but, due to the nature of rigid body motions, necessarily depends on the moving frame, despite being an objective description in the sense of [35]. However, our experimental protocol explicitly requires the subject to grasp the tool at a prescribed position, i.e. at fixed distance 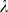 from the tip as in Fig. 1. Arguably, this induces a unique, natural choice for the position of the moving frame 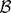. It should be noted that *our analysis only depends on the position of the moving frame and not on its orientation*, as shown in supporting information Text S1. Therefore, although mathematically there might be a general dependence on the choice of the body frame, a well defined task should always induce a natural choice of such a frame. This is consistent with the idea that unambiguously defined tasks should allow to measure performance in an unambiguous way, as also observed by Sternad et al. [5].

### Possible generalizations

#### Dynamic tasks

In this paper, we focused on static pointing tasks. However, starting from the original work by Scholz and Schoner [4] the classical UCM concept was applied to a variety of dynamic tasks, including sit-to-stand transitions, reaching and walking. In these works, the UCM method was applied to trajectories during consecutive movements which were re-scaled in time and aligned so that corresponding points occurring at the same normalized time could be sampled [4]. The only difference of data analysis between static and dynamic tasks is in the way samples are collected. In the static task, the samples are consecutive postures within a specific trial. In dynamic tasks, an *ensemble* of trials is considered and, at a specific normalized time, one posture per trial is sampled. The analysis is then repeated at different time slices.

In general, this approach might raise concerns in dynamic tasks which are not appropriately timed. When a task is self-paced, there is no guarantee that events occurring in different trials at the same normalized time are necessarily related.

In any case, the method would still be applicable to *tracking tasks* where, repeatedly across trials, a subject is asked to track a visible target which evolves along a predefined path at a predefined speed (the whole path might or might not be displayed). At any given time, the subject is supposed to be on target. Therefore, postures at that very time could be sampled across trials and used in the UCM analysis, as for a static task.

#### Visually perceived geometry

In a recent study, Danziger and Mussa-Ivaldi [16] asked subjects to control a simulated 2-link planar arm via a (hyper-redundant) map from finger postures to the two joint angles of the linkage. According to the type of visual feedback (one group of subjects could see the whole linkage while another group could only see the moving end-tip), subjects would move along paths which could be associated with the geodesics of the visually perceived tool, i.e. either a point or an articulated linkage. These findings are consistent with the hypothesis that, from visual observations, the brain can infer (geo)metric properties of tools and can adapt to produce controlled motions consistent with the perceived metric properties (e.g. moving along shortest paths).

#### Intrinsic statistics

As a final note, the type of statistical analysis needs not to be restricted to simple analysis of variance, as done in this paper along the line of classical UCM approach. Once a Riemannian framework is in place, as in our paper or in the work of Biess et al. [8], [9], one is given a host of computational tools to perform intrinsic statistics, as recently presented for example in the field of medical imaging [18].

## Supporting Information

Text S1Our analysis does not depend on the orientation of the moving frame 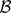.(PDF)
