# Supplementary material for: Analysis of Accuracy in Pointing with Redundant Hand-held Tools: A Geometric Approach to the Uncontrolled Manifold Method
Source: PLoS Comput Biol. 2013 Apr 4;9(4):e1002978. doi: 10.1371/journal.pcbi.1002978 (PMC3617015; doi:10.1371/journal.pcbi.1002978)
Supplement: Text S1 — Our analysis does not depend on the orientation of the moving frame . (PDF) [file pcbi.1002978.s001.pdf]

Text S1

Our analysis does not depend on the orientation of the moving frame $\mathcal{B}$

In this section we show that our analysis of variance, in particular eq. (35), does not depend on the orientation of the moving frame $\mathcal{B}$. In particular, we will show that all the vector quantities used in our formulation, when expressed with respect to (w.r.t.) a new, rotated moving frame will simply be rotated by the same matrix defining the rotation of the new moving frame w.r.t. to the old one. Eventually, computing variance in eq.(35) will only involve inner products between such vectors, which we will show to be invariant to rotations. Intuitively, the angle between two vectors does not change if both vectors undergo the same rotation.

Consider a new moving frame $\mathcal{B}'$, with new axes $\{\boldsymbol{c}'_1, \boldsymbol{c}'_2, \boldsymbol{c}'_3\}$ which are related to the old axes via a fixed, arbitrary, constant rotation R_x. Hereafter, we shall adopt the following naming conventions: different names will denote different vectors; upper-case names will denote vectors with coordinates w.r.t the fixed frame $\mathcal{S}$; for lower-case names, the 'prime' symbol ($'$) will denote coordinates w.r.t. $\mathcal{B}'$, otherwise coordinates are w.r.t. $\mathcal{B}$. For example, $\boldsymbol{c}'_3$ and $\boldsymbol{e}_3$ are *different* vectors but both happen to have coordinates $[0\ 0\ 1]^T$ when expressed w.r.t $\mathcal{B}'$ and $\mathcal{B}$, respectively. On the other hand, the *same* vector $\boldsymbol{e}_3 = [0\ 0\ 1]^T$ (aligned with the tool axis, by definition) can be represented w.r.t $\mathcal{B}'$ by $\boldsymbol{e}'_3 = R_x^T \boldsymbol{e}_3$, i.e. the third column of the matrix R_x^T.

Therefore a tool pose, described by $g = \{R, \boldsymbol{P}_{grip}\}$ w.r.t. the old moving frame $\mathcal{B}$, would now be described as $g' = \{R', \boldsymbol{P}_{grip}\}$ w.r.t. $\mathcal{B}'$ where

$$R' = R R_x \quad \longleftrightarrow \quad R = R' R_x^T$$

Note that the position coordinates $\boldsymbol{P}_{grip}$ are expressed w.r.t to the fixed frame $\mathcal{S}$, therefore would be unaffected by a change of moving frame.

Eq. (24)–(25) are still valid but, if rewritten in terms of R', would now be

$$\boldsymbol{P}_{tip} = \boldsymbol{P}_{grip} - \lambda\, R\boldsymbol{e_3} = \boldsymbol{P}_{grip} - \lambda\, R'\, R_x^T \boldsymbol{e_3}$$

$$\boldsymbol{V}_{tip} = \boldsymbol{V}_{grip} - \lambda\ \dot{R}\boldsymbol{e_3} = \boldsymbol{V}_{grip} - \lambda\ \dot{R}'\, R_x^T \boldsymbol{e_3}.$$

Expressing the velocities in *new* body coordinates $\boldsymbol{v}'_{tip}$ and $\boldsymbol{v}'_{grip}$, via the transformations $\boldsymbol{V}_{tip} = R'\,\boldsymbol{v}'_{tip}$ and $\boldsymbol{V}_{grip} = R'\,\boldsymbol{v}'_{grip}$, we obtain

$$\boldsymbol{v}'_{tip} = \boldsymbol{v}'_{grip} - \lambda\ \boldsymbol{\omega}' \times \left(R_x^T \boldsymbol{e_3}\right)$$

or

$$\boldsymbol{v}'_{tip} = J' \begin{bmatrix} \boldsymbol{v}'_{grip} \\ \boldsymbol{\omega}' \end{bmatrix}$$

where the new Jacobian J' is as follows (I is the 3×3 identity matrix):

$$J' = \begin{bmatrix} I & \lambda\,(\widehat{R_x^T \boldsymbol{e_3}}) \end{bmatrix}$$

We shall maintain the *same metric form* of eq.(19) because the only parameter defining the metric λ is *unchanged* as we are considering a new moving frame $\mathcal{B}'$ which has the *same origin* as the old moving frame $\mathcal{B}$, i.e. the same distance from the tool-tip.

The null space can be computed in the new moving frame coordinates by imposing $0 = J'[\boldsymbol{v}'\ \boldsymbol{\omega}']$. The following vectors form a basis for the null space in the new coordinates

$$\boldsymbol{n}'_j = \begin{bmatrix} R_x^T & 0 \\ 0 & R_x^T \end{bmatrix} \boldsymbol{n}_j$$

where $j = 1, 2, 3$. This can be verified by checking that $J'\boldsymbol{n}'_j = R_x^T(J\boldsymbol{n}_j) = 0$, for $j = 1, 2, 3$. Similarly, we can verify that the following vectors consitute a basis for the orthogonal complement

$$\boldsymbol{o}'_j = \begin{bmatrix} R_x^T & 0 \\ 0 & R_x^T \end{bmatrix} \boldsymbol{o}_j$$

This means that the null space and the orthogonal space are *unchanged*, although the coordinates of their basis vectors are different (all rotated by R_x^T) simply because expressed with respect to another reference frame.

Remark: a different metric with $\lambda' \neq \lambda$ would have led to a different orthogonal complement, while the null space would have been unchanged as it does not depend on the metric (however, the 'length' of its vectors would have been affected by the new scale λ').

When computing the mean pose via equation of (32), it is clear that the mean position (first equation) is not affected by the orientation of the moving frame. As for the mean orientation, suppose that we compute the average rotation R_0 for a sequence R_i expressed with respect to the old moving frame via the second of (32). At the same time, for the *same* sequence of physical positions of the tool, imagine we compute the mean rotation R'_0 from poses w.r.t. the new body frame $\mathcal{B}'$, corresponding to a sequence of rotations $R'_i = R_i R_x$. By means of the following properties (see [19])

$$\begin{aligned} \|A \log_\vee(B)\| &= \|\log_\vee(B)\| & \text{(S1.1)} \\ \log_\vee(ABA^T) &= A \log_\vee(B) & \text{(S1.2)} \end{aligned}$$

where A and B are rotation matrices, the following algebraic manipulations can be derived:

$$\begin{aligned} R'_0 &= \underset{R'}{\text{argmin}} \sum_{i=1}^{N} \|\log_\vee(R_i'^T R')\|^2 \\ &\overset{(S1.1),(S1.2)}{=} \underset{R'}{\text{argmin}} \sum_{i=1}^{N} \|\log_\vee(R_x R_i'^T R' R_x^T)\|^2 \\ &= \underset{R'}{\text{argmin}} \sum_{i=1}^{N} \|\log_\vee((R'_i R_x^T)^T R' R_x^T)\|^2 \\ &= \left(\underset{R' R_x^T}{\text{argmin}} \sum_{i=1}^{N} \|\log_\vee((R'_i R_x^T)^T R' R_x^T)\|^2 \right) R_x \\ &= \left(\underset{R}{\text{argmin}} \sum_{i=1}^{N} \|\log_\vee(R_i^T R)\|^2 \right) R_x \\ &= R_0 R_x \end{aligned}$$

The deviations from the mean can then be computed as

$$\boldsymbol{\Delta}'_i = \begin{bmatrix} R_0'^T(\boldsymbol{P}_i - \boldsymbol{P}_0) \\ \log_\vee(R_0'^T R'_i) \end{bmatrix} = \begin{bmatrix} R_x^T R_0^T(\boldsymbol{P}_i - \boldsymbol{P}_0) \\ \log_\vee(R_x^T R_0^T R_i R_x) \end{bmatrix} = \begin{bmatrix} R_x^T R_0^T(\boldsymbol{P}_i - \boldsymbol{P}_0) \\ R_x^T \log_\vee(R_0^T R_i) \end{bmatrix} = \begin{bmatrix} R_x^T & 0 \\ 0 & R_x^T \end{bmatrix} \boldsymbol{\Delta}_i$$

Therefore, deviations $\boldsymbol{\Delta}'_i$ as well as null vectors $\boldsymbol{n}'_i$ and orthogonal complement $\boldsymbol{o}'_i$ represent the *same* vectors as $\boldsymbol{\Delta}_i, \boldsymbol{n}_i, \boldsymbol{o}_i$ (respectively), simply with coordinates rotated by R_x^T, due to the change of moving frame. As the inner product (20) is invariant to rotations, the projections in eq.(34) as well as equation (35) are invariant to rotations of the moving frame.

Q.E.D.
